# Second primary colorectal cancer in adults: a SEER analysis of incidence and outcomes

**DOI:** 10.1186/s12876-023-02893-2

**Published:** 2023-07-26

**Authors:** Weijian Lun, Canhua Luo

**Affiliations:** Gastroenterology department of The Sixth Affiliated Hospital, School of Medicine, South China University of Technology, 120# Guidan Road, Nanhai District, Foshan, 528200 Guangdong Province China

**Keywords:** Colorectal cancer, Second primary colorectal cancer, Influencing factors, Initial primary colorectal cancer, Survival

## Abstract

**Background:**

At present, there was no large epidemiological study exploring the actual incidence and survival of second primary colorectal cancer (spCRC). The different characteristics and survival of patients with spCRC and initial primary colorectal cancer (ipCRC) still need to be elucidated. In addition, the factors leading to different survival status of spCRC and ipCRC were still unclear. Our study plan to explore the annual incidence trend of spCRC as well as the factors influencing the occurrence and survival outcome of spCRC.

**Methods:**

This cohort study analyzed the data of 4680 spCRC patients and 330,937 initial primary colorectal cancer (ipCRC) patients. Whether patients had spCRC and whether spCRC patients survived or died were regarded as outcomes. The annual incidence of spCRC from 2004 to 2016 was analyzed by Jointpoint regression analysis. The truncation points were found, and the annual percentage change (APC) of each segment was calculated to explore the trend of spCRC change in the United States. Univariate and multivariable cox regression analyses were conducted to identify factors associated with the occurrence and prognosis of spCRC patients.

**Results:**

The total incidence of spCRC was decreased during 2000–2016 on the whole. The overall incidence of spCRC was lowered in both males and females despite 2013–2014, in the left colon, right colon, rectum and others. The incidence of spCRC was decreased in both 18–49 years’ people and ≥ 50 years’ people during 2000–2016, and the incidence of spCRC in the ≥ 50 years’ people group was higher than those of 18–49 years. Insured (OR = 0.867 (0.778–0.966), initial primary site of other digestive (OR = 0.46, 95%CI: 0.42–0.50), rectum (OR = 0.74, 95%CI: 0.66–0.82), or right colon (OR = 0.73, 95%CI: 0.68–0.79), N 1 stage (OR = 0.87, 95%CI: 0.76–0.99), M 1 stage (OR = 0.49, 95%CI: 0.30–0.80), AJCC II stage (OR = 0.70, 95%CI: 0.60–0.82), AJCC III stage (OR = 0.69, 95%CI: 0.56–0.84), and radiation (OR = 0.69, 95%CI: 0.57–0.83) were associated with the risk of spCRC. At the end of follow-up, 2,246 spCRC patients were survived and 2,434 spCRC patients were dead. Patients with spCRC had poor survival probability than patients with ipCRC. Older age (HR = 1.02, 95%CI: 1.02–1.03), male (HR = 1.13, 95%CI: 1.04–1.23), Black (HR = 1.20, 95%CI: 1.06–1.35), uninsured (HR = 1.36, 95%CI: 1.16–1.59), Signet ring cell carcinoma (HR = 1.64, 95%CI: 1.19–2.25), T4 stage (HR = 1.63, 95%CI: 1.32–2.01), N2 stage (HR = 1.36, 95%CI: 1.08–1.72), M1 stage (HR = 4.51, 95%CI: 2.00–10.18), AJCC III (HR = 1.47, 95%CI: 1.08–1.98), and radiation (HR = 1.82, 95%CI: 1.43–2.33) were associated with increased risk of mortality in spCRC patients.

**Conclusion:**

The incidence of spCRC was decreased except in people with initial primary tumor grade IV and those aged 15–39 years. The overall survival of spCRC patients was lower than ipCRC patients. Cancer patients with older age, high tumor grade, TNM stage, and AJCC stage should be caution to the occurrence of spCRC and timely interventions should be provided for spCRC patients to improve their outcomes.

**Supplementary Information:**

The online version contains supplementary material available at 10.1186/s12876-023-02893-2.

## Background

Colorectal cancer (CRC) is the third most common malignancy and ranks the third in cancer-associated mortality all over the world [1]. In 2020, 1.93 million new CRC cases were diagnosed, and 0.94 million patients were died due to CRC, which accounted for 10% of the global cancer incidence and 9.4% of all cancer caused deaths [2]. In some recent studies, the incidence and risk of second primary malignancies (SPM) were reported to be increased [3, 4], this may be due to that the early detection and treatments in cancer patients were improved and the life expectancy of cancer patients was extended [5]. The risk of a SPM is higher in patients with cancer in remission than a population with no history of cancer with about 17%-19% cancer patients were SPMs [6, 7]. Second primary colorectal cancer (spCRC) is one of the SPMs which was frequently diagnosed in cancer survivors [8]. Patients with spCRC were reported to have worse outcome than those with initial primary CRC (ipCRC) [9]. To identify the incidence and the mortality as well as their influencing factors were essential for better management of spCRC patients.

Presently, there were some studies analyzed the incidence or survival of spCRC in initial primary CRC patients and found that previous CRC was a risk factor for spCRC [10, 11]. Pruitt et al. conducted a study to explore the survival of newly diagnosed CRC patients with a history of previous cancer [12]. Some studies only analyzed the incidence and survival as well as the influencing factors of spCRC in ipCRC patients, but patients with spCRC developing from other types of initial primary malignancies were not analyzed. Other studies explored the incidence and risk factors for any second primary cancers in patients with ipCRC [11, 13, 14]. Bae et al. conducted a multi-center study evaluated the risk of spCRC in patients with a prior history of prostate, breast or lung cancer [15]. At present, there was no large epidemiological study on exploring the actual incidence and survival of spCRC. The different characteristics and survival of patients with spCRC and ipCRC still need to be elucidated. In addition, the factors leading to different survival status of spCRC and ipCRC were still unclear.

In our study, the purpose was to explore the annual incidence trend of spCRC as well as the incidence trend of spCRC in different subgroups of age, grade, site and gender. The characteristics of initial primary cancers were analyzed in spCRC patients to identify factors influencing the occurrence of spCRC. The characteristics and survival of spCRC and ipCRC patients were compared, and the factors influencing the survival outcome of spCRC were analyzed.

## Methods

### Study design and population

**Fig. 1:**
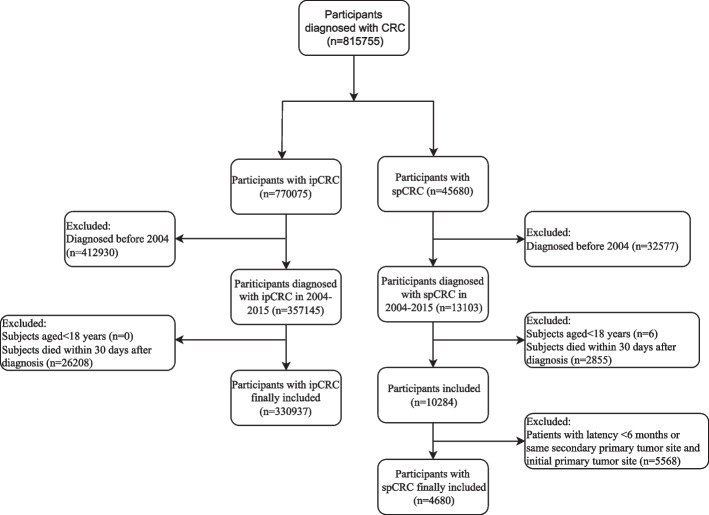
The screen process of the participants

This cohort study included 815,755 patients diagnosed with CRC based on the International Classification of Diseases for Oncology (ICD-O)-3 from Surveillance, Epidemiology, and End Results (SEER) Program (www.seer.cancer.gov) SEER*Stat Database. Among them, 45,680 were spCRC patients and 770,075 were ipCRC patients. The SEER database is a population-based cancer reporting system conducted by the National Cancer Institute covering about 28% of the total population in the United States. Researchers have free access to the database and can utilize the data to explore cancer epidemiology and outcomes [16]. The incidence of spCRC was analyzed in spCRC patients from 2000–2016 in SEER database. We analyzed the data of CRC patients in SEER database from 2004–2015 to explore the influencing factors associated with the occurrence and survival of spCRC patients. Those who diagnosed before 2004 were excluded as the 6^th^ version of the American Joint Committee on Cancer (AJCC) TNM stage was applied in SEER from 2004. In total, 45,680 spCRC patients were involved in our study, after excluding patients before 2004 (*n* = 32,577), patients aged < 18 years (*n* = 6), those died within 30 days after a confirmed diagnosis (*n* = 2,855) and people with latency < 6 months or same secondary primary tumor site and the initial primary tumor site (*n* = 5,568), and 4680 spCRC patients were finally included. The total sample of ipCRC extracted from SEER database were 770,075, and 412,930 patients diagnosed before 2004 and 26,208 patients died within 30 days were excluded. Finally, 330,937 ipCRC patients were involved in (Fig. 1). The requirement of ethical approval for this was waived by the Institutional Review Board of The Sixth Affiliated Hospital, School of Medicine, South China University of Technology, because the data was accessed from SEER (a publicly available database). All individuals provided written informed consent before participating in the study. All methods were carried out in accordance with relevant guidelines and regulations (declaration of Helsinki).

### Variables

The varaibles in spCRC patients included age, gender, race (White, Black or other/unknown), insurance (any Medicaid, insured, insured/no specifics, uninsured or unknown), initial primary tumor site (left colon, other digestive system, rectum, or right colon), grade [Well differentiated (grade I, moderately differentiated (grade II), poorly differentiated (grade III), undifferentiated or anaplastic (grade IV) or unknown], T stage (T0/T1/T2, T3, T4 or unknown), N stage (N0, N1, N2, N3 or NX), M stage (M0, M1, or MX), AJCC (0, I, II, III, IV or no/unknown), surgery (yes or no/unknown), radiation (yes or no/unknown), chemotherapy (yes, no/unknown), second primary tumor site (right colon, left colon, rectum or others), radiation sequence (no radiation and/or no surgery, after surgery, before and after surgery, before surgery or unknown), and histology (adenocarcinoma, mucous adenocarcinoma, signet ring cell carcinoma or others).

Variables analyzed in ipCRC patients included age, gender, race (White, Black or other/unknown), insurance (any Medicaid, insured, insured/no specifics, uninsured or unknown), initial primary tumor site (left colon, other digestive system, rectum, right colon, or other gastrointestinal tract cancers), grade [Well differentiated (grade I, moderately differentiated (grade II), poorly differentiated (grade III), undifferentiated or anaplastic (grade IV) or unknown], T stage (T0/T1/T2, T3, T4 or unknown), N stage (N0, N1, N2, N3 or unknown), M stage (M0, M1, or unknown), AJCC (0, I, II, III, IV or unknown stage), radiation (yes or no/unknown), chemotherapy (yes, no/unknown), and histology (adenocarcinoma, mucous adenocarcinoma, signet ring cell carcinoma or others).

### Outcome variables

Whether patients had spCRC and whether spCRC patients survived or died were regarded as outcomes. When the outcome was whether spCRC occurred, the follow-up was ended up when those diagnosed with spCRC. When the outcome was whether spCRC patients survived or died, the follow-up was ended when the patients died. All follow-up was ended in April 2019.

### Definition of spCRC

Primary gastrointestinal tract cancers including left colon cancer, right colon cancer, rectum cancers, other digestive cancers, and other gastrointestinal tract cancers were identified according to the cancer site of origin, date of diagnosis, histology, tumor behavior (i.e., in situ versus invasive), and laterality of paired organs in the SEER database. SPMs occurring two or more months after the initial diagnosis were considered as separate primaries unless the medical record stated that the tumor was recurrent or metastatic [17]. In the current study, the definition of spCRC fulfilled the following criteria: (1) interval between the diagnosis of initial primary cancers and spCRC (latency) ≥ 6 months; (2) difference in the primary site between initial primary cancers and spCRC; (3) difference in histology if the primary site is the same as the primary site of the initial primary cancers.

### Statistical analysis

The normality of the data was assessed via by Shapiro test. The continuous variables of normal distribution were expressed as Mean ± standard deviation (Mean ± SD), and the t-test was used for comparison between groups. Non-normally distributed measurement data were represented by median and quartile spacing [M (Q_1_, Q_3_)], and Mann–Whitney U test was used for comparison between groups. Enumeration data were described as the number of cases and composition ratio [n (%)], and comparison between groups was performed by χ^2^ test or Fisher’s exact probability method. The annual incidence of spCRC from 2004 to 2016 was analyzed by Joinpoint regression analysis. The truncation points were found, and the annual percentage change (APC) of each segment was calculated to explore the trend of spCRC change in the United States. Univariate and multivariable logistic regression analyses were to identify factors associated with the occurrence of spCRC patients, and variables with statistical difference in the univariate logistic regression analysis were included in the multivariable logistic regression analysis [18]. Univariate and multivariable cox regression analyses were to identify factors associated with the prognosis of ipCRC and spCRC patients. Statistically significant variables in univariate cox regression analysis were included in the multivariable cox regression model. All statistical analysis was completed using SAS v9.4, and trend analysis was completed using Joinpoint Regression Analysis v.4.6.0.0.

## Results

### The epidemiological trends of spCRC incidence

**Fig. 2:**
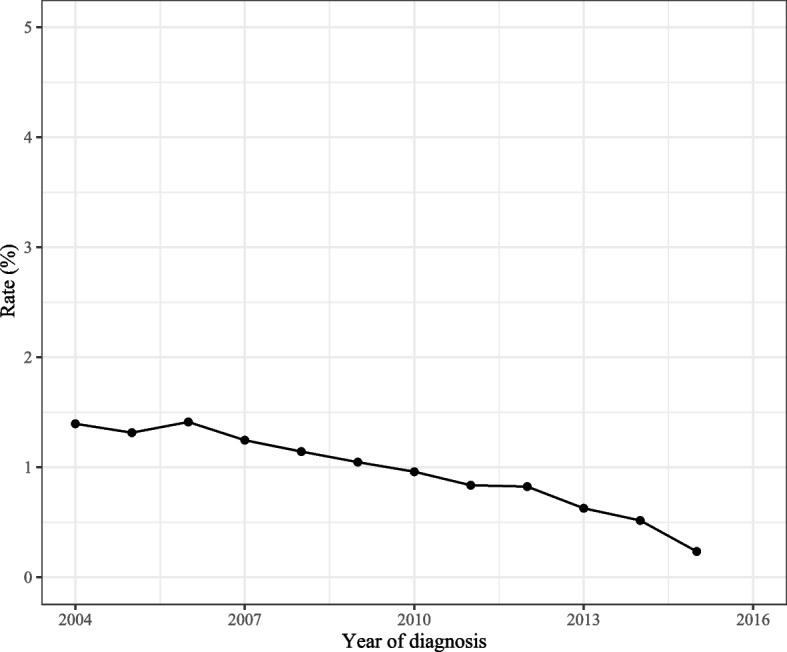
The incidence of spCRC in all participants

**Fig. 3:**
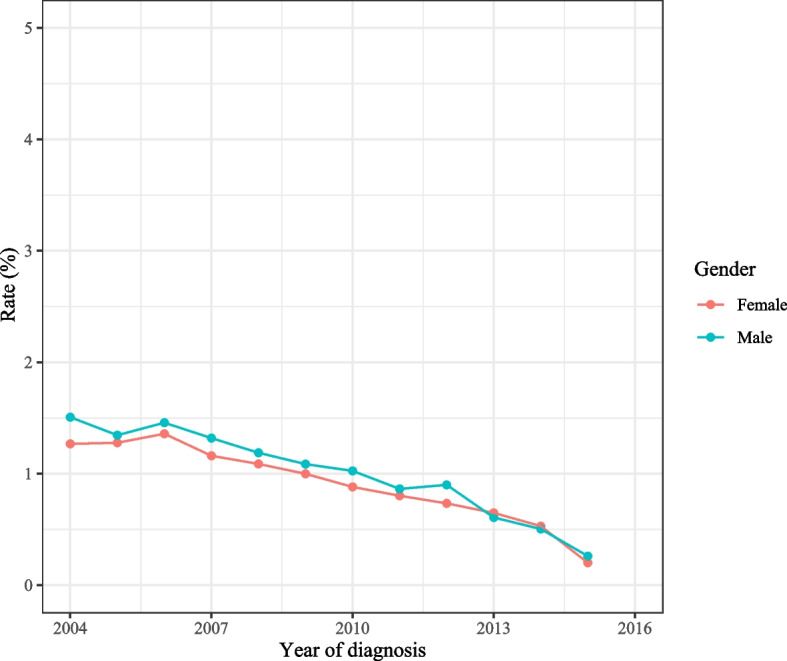
The incidences of spCRC in different genders

**Fig. 4:**
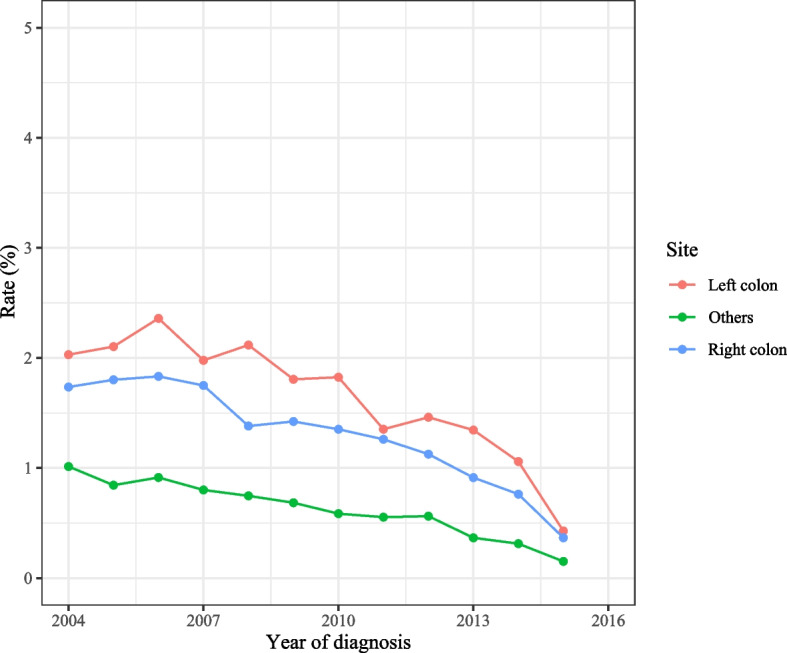
The incidences of spCRC in different tumor sites

**Fig. 5:**
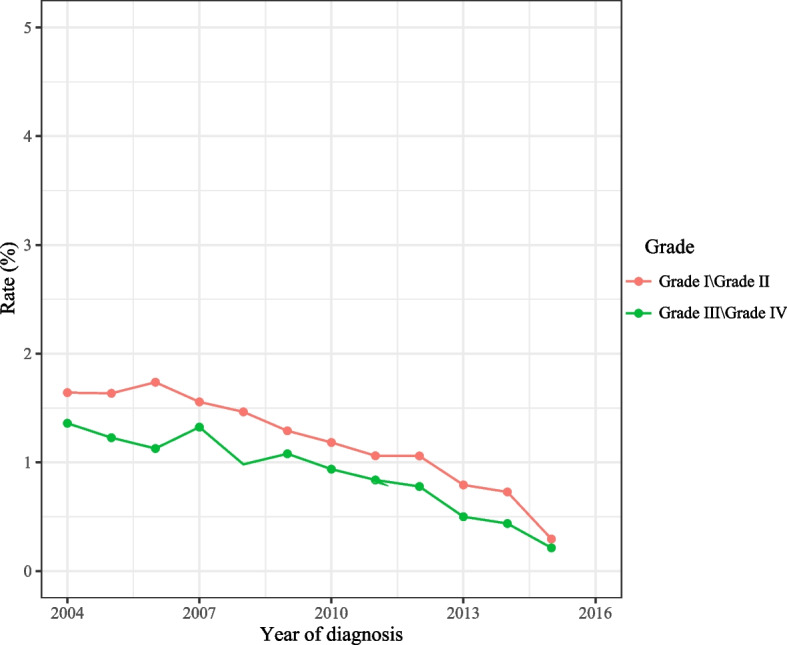
The incidences of spCRC in different tumor grade

**Fig. 6:**
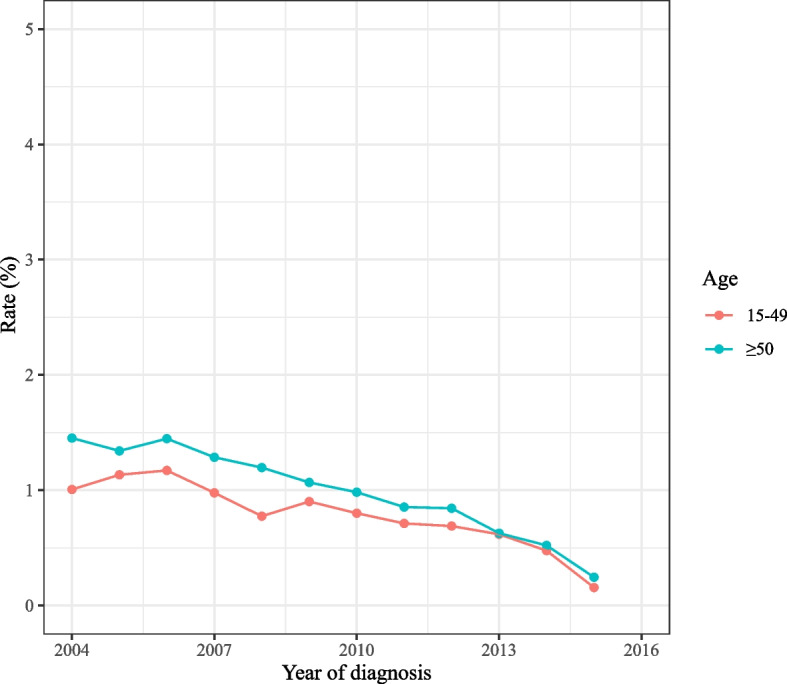
The incidences of spCRC in different ages

As observed in Fig. 2, the total incidence of spCRC was decreased during 2000–2016 on the whole. The overall incidence of spCRC was lowered during 2000–2016 in both males and females, the incidences of spCRC in males were higher than females despite 2013–2014 (Fig. 3). The decreased incidences of spCRC were observed in the left colon, right colon, and others, and the most frequently diagnosed site of spCRC was the left colon during 2000–2016 (Fig. 4). As for different tumor grade, a decrease of incidence of spCRC was observed in well differentiated (grade I) and moderately differentiated (grade II). The slowly reduced incidences of spCRC were identified in poorly differentiated (grade III), and undifferentiated or anaplastic (grade IV) group during 2006–2007 (Fig. 5). The incidence of spCRC was decreased in both 18–49 years’ people and ≥ 50 years’ people during 2000–2016, and the incidence of spCRC in the ≥ 50 years’ people group was higher than those of 18–49 years (Fig. 6). The initial primary tumor sites of spCRC patients were exhibited in Supplementary Fig. 1. The right colon cancer showed the highest proportion in all initial primary tumors [1428 (31.67%)] followed by left colon cancer [1389 (29.68%)].

### Factors associated with the occurrence of spCRC

In the adjusted logistical regression Model 1, age, gender, insurance, primary tumor site, histology, grade, T stage, N stage, M stage, AJCC stage, radiation, chemotherapy and radiation sequence might be factors associated with the occurrence of spCRC. In Model 2, these variables were included and the data of stepwise regression revealed that insured (OR = 0.867 (0.778–0.966), initial primary site of other digestive (OR = 0.46, 95%CI: 0.42–0.50), rectum (OR = 0.74, 95%CI: 0.66–0.82), or right colon (OR = 0.73, 95%CI: 0.68–0.79), N1 stage (OR = 0.87, 95%CI: 0.76–0.99), M1 stage (OR = 0.49, 95%CI: 0.30–0.80), AJCCII stage (OR = 0.70, 95%CI: 0.60–0.82), AJCCIII stage (OR = 0.69, 95%CI: 0.56–0.84), and radiation (OR = 0.69, 95%CI: 0.57–0.83) were associated with decreased risk of spCRC. Age (OR = 1.01, 95%CI: 1.01–1.01), male (OR = 1.17, 95%CI: 1.10–1.24), mucous adenocarcinoma (OR = 1.19, 95%CI: 1.07–1.33), T3 stage (OR = 1.61, 95%CI: 1.40–1.85), T4 stage (OR = 1.90, 95%CI: 1.63–2.22), and radiation after surgery (OR = 1.52, 95%CI: 1.22–1.88) were associated with higher risk of spCRC (Table 1).

**Table 1:** Factors associated with the occurrence of spCRC

Variables	OR (95%CI)	*P*	OR (95%CI)	*P*
Age	1.01 (1.01–1.01)	< 0.001	1.01 (1.01–1.01)	< 0.001
Sex				
Female	Ref		Ref	
Male	1.09 (1.03–1.15)	0.004	1.17 (1.10–1.24)	< 0.001
Race				
Black	Ref			
Unknown	0.91 (0.80–1.03)	0.126	0.91 (0.80–1.03)	0.133
White	1.02 (0.93–1.11)	0.743	0.95 (0.87–1.04)	0.257
Insurance				
Any Medicaid	Ref		Ref	
Insured	0.96 (0.86–1.07)	0.463	0.87 (0.78–0.97)	0.010
Insured/No specifics	1.12 (0.99–1.28)	0.083	0.98 (0.86–1.12)	0.797
Uninsured	1.10 (0.89–1.36)	0.387	1.09 (0.88–1.35)	0.453
Blank(s)	1.53 (1.37–1.70)	< 0.001	1.37 (1.29–1.53)	< 0.001
Tumor site				
Left colon	Ref		Ref	
Other cancer	0.22 (0.03–1.54)	0.127	0.79 (0.11–5.66)	0.814
Other digestive	0.30 (0.27–0.32)	< 0.001	0.46 (0.42–0.50)	< 0.001
Other rectum	0.53 (0.49–0.59)	< 0.001	0.74 0.66–0.82)	< 0.001
Right colon	0.78 (0.73–0.84)	< 0.001	0.73 (0.68–0.79)	< 0.001
Histology				
Adenocarcinoma	Ref			
Mucous adenocarcinoma	1.27 (1.14–1.42)	< 0.001	1.19 (1.07–1.33)	0.002
Signet ring cell carcinoma	0.55 (0.42–0.71)	< 0.001	0.91 (0.70–1.20)	0.481
Others	0.49 (0.45–0.52)	< 0.001	0.85 (0.78–0.93)	< 0.001
Grade				
Grade I/Grade II	Ref			
Grade III/Grade IV	0.76 (0.70–0.82)	< 0.001	0.92 (0.84–0.99)	0.039
Unknown	0.41 (0.38–0.45)	< 0.001	0.73 (0.66–0.80)	< 0.001
T stage				
T2	Ref			
T3	1.32 (1.23–1.41)	< 0.001	1.61 (1.40–1.85)	< 0.001
T4	1.24 (1.12–1.36)	< 0.001	1.90 (1.63–2.22)	< 0.001
Unknown	0.53 (0.49–0.59)	< 0.001	1.28 (1.01–1.61)	0.040
N stage				
N0	Ref			
N1	0.75 (0.70–0.81)	< 0.001	0.87 (0.76–0.99)	0.044
N2	1.08 (0.98–1.19)	0.108	1.09 (0.93–1.27)	0.312
N3	0.30 (0.14–0.64)	0.002	0.71 (0.32–1.57)	0.394
Unknown	0.34 (0.31–0.38)	< 0.001	0.78 (0.62–0.98)	0.036
M stage				
M0	Ref			
M1	0.40 (0.36–0.44)	< 0.001	0.49 (0.30–0.80)	0.004
Unknown	0.34 (0.30–0.38)	< 0.001	0.53 (0.41–0.68)	< 0.001
AJCC stage				
I	Ref			
II	1.12 (1.03–1.21)	0.005	0.70 (0.60–0.82)	< 0.001
III	1.05 (0.96–1.13)	0.279	0.69 (0.56–0.84)	0.001
IV	0.42 (0.38–0.48)	< 0.001	0.66 (0.41–0.08)	0.090
Unknown	0.46 (0.41–0.51)	< 0.001	0.83 (0.64–1.06)	0.138
Radiation				
No/Unknown	Ref			
Yes	0.68 (0.62–0.74)	< 0.001	0.69 (0.57–0.83)	< 0.001
Chemotherapy				
No/Unknown	Ref			
Yes	0.70 (0.66–0.74)	< 0.001	0.93 (0.86–1.00)	0.055
Radiation sequence				
Only one	Ref		Ref	
After	0.93 (0.82–1.05)	0.252	1.52 (1.22–1.88)	0.001
Before	0.72 (0.63–0.84)	< 0.001	0.95 (0.76–1.20)	0.687
Both	0.46 (0.21–1.03)	0.059	0.64 (0.28–1.46)	0.288
Unknown	1.04 (0.39–2.78)	0.940	1.59 (0.59–4.28)	0.358

*spCRC* second primary colorectal cancer, *AJCC* American Joint Committee on Cancer, *RR* risk ratio, *CI* confidence interval

Model 1 Univariate cox regression analysis

Model 2 Multivariable cox regression analysis including variables with statistical difference in Model 1

### The characteristics of spCRC patients

At the end of follow-up, 2,246 spCRC patients were survived and 2,434 spCRC patients were dead. The characteristics of patients in the survival group and death group were presented in Table 2. The mean age of the survival group was lower than the death group (64.33 years vs 67.99 years). The proportion of patients received radiation (310.69% vs 14.17%) or chemotherapy (39.15% vs 32.81) for the initial primary tumor in the survival group was lower than the death group. The distributions of patients with different race, insurance, tumor site, tumor histology, grade, T stage, N stage, M stage, AJCC stage, radiation sequence were statistically different in the survival group and death group.

**Table 2:** The baseline characteristics of spCRC patients survived or dead

		Groups			
Variables	Total (*n* = 4680)	Survived (*n* = 2246)	Dead group (*n* = 2434)	Statistics	*P*
Age, Mean ± SD	66.24 ± 12.76	64.33 ± 12.57	67.99 ± 12.68	t = -9.91	< 0.001
Sex, n(%)				χ^2^ = 0.771	0.380
Female	2061 (44.04)	1004 (44.70)	1057 (43.43)		
Male	2619 (55.96)	1242 (55.30)	1377 (56.57)		
Race, n (%)				χ^2^ = 11.400	0.003
Black	564 (12.05)	253 (11.26)	311 (12.78)		
Unknown	474 (10.13)	260 (11.58)	214 (8.79)		
White	3642 (77.82)	1733 (77.16)	1909 (78.43)		
Insurance, n (%)				χ^2^ = 101.191	< 0.001
Any Medicaid	410 (8.76)	207 (9.22)	203 (8.34)		
Blank	1694 (36.20)	650 (28.94)	1044 (42.89)		
Insured	1930 (41.24)	1052 (46.84)	878 (36.07)		
Insured/No specifics	539 (11.52)	281 (12.51)	258 (10.60)		
Uninsured	107 (2.29)	56 (2.49)	51 (2.10)		
Histology, n (%)				χ^2^ = 25.108	< 0.001
Adenocarcinoma	3320 (70.94)	1543 (68.70)	1777 (73.01)		
Mucous adenocarcinoma	377 (8.06)	181 (8.06)	196 (8.05)		
Signet ring cell carcinoma	60 (1.28)	19 (0.85)	41 (1.68)		
Others	923 (19.72)	503 (22.40)	420 (17.26)		
Grade, n (%)				χ^2^ = 16.618	< 0.001
Grade I/Grade II	3250 (69.44)	1598 (71.15)	1652 (67.87)		
Grade III/Grade IV	761 (16.26)	314 (13.98)	447 (18.36)		
Unknown	669 (14.29)	334 (14.87)	335 (13.76)		
T stage, n (%)				χ^2^ = 76.008	< 0.001
T2	1430 (30.56)	798 (35.53)	632 (25.97)		
T3	2057 (43.95)	926 (41.23)	1131 (46.47)		
T4	588 (12.56)	214 (9.53)	374 (15.37)		
Unknown	605 (12.93)	308 (13.71)	297 (12.20)		
N stage, n (%)				χ^2^ = 80.725	< 0.001
N0	2871 (61.35)	1501 (66.83)	1370 (56.29)		
N1	881 (18.82)	363 (16.16)	518 (21.28)		
N2	519 (11.09)	176 (7.84)	343 (14.09)		
N3	7 (0.15)	3 (0.13)	4 (0.16)		
Unknown	402 (8.59)	203 (9.04)	199 (8.18)		
M stage, n (%)				χ^2^ = 71.790	< 0.001
M0	3982 (85.09)	1964 (87.44)	2018 (82.91)		
M1	339 (7.24)	89 (3.96)	250 (10.27)		
Unknown	359 (7.67)	193 (8.59)	166 (6.82)		
AJCC stage, n (%)				χ^2^ = 129.666	< 0.001
I	1247 (26.65)	716 (31.88)	531 (21.82)		
II	1396 (29.83)	686 (30.54)	710 (29.17)		
III	1150 (24.57)	465 (20.70)	685 (28.14)		
IV	360 (7.69)	103 (4.59)	257 (10.56)		
Unknown	527 (11.26)	276 (12.29)	251 (10.31)		
Radiation, n (%)				χ^2^ = 12.997	< 0.001
No/Unknown	4095 (87.50)	2006 (89.31)	2089 (85.83)		
Yes	585 (12.50)	240 (10.69)	345 (14.17)		
Chemotherapy, n (%)				χ^2^ = 20.350	< 0.001
No/Unknown	2990 (63.89)	1509 (67.19)	1481 (60.85)		
Yes	1690 (36.11)	737 (32.81)	953 (39.15)		
Radiation sequence, n (%)				-	0.004
Only one	4217 (90.11)	2044 (91.01)	2173 (89.28)		
After surgery	261 (5.58)	98 (4.36)	163 (6.70)		
Before surgery	192 (4.10)	97 (4.32)	95 (3.90)		
Both	6 (0.13)	4 (0.18)	2 (0.08)		
unknown	4 (0.09)	3 (0.13)	1 (0.04)		

*spCRC* second primary colorectal cancer, *ipCRC* initial primary colorectal cancer, *AJCC* American Joint Committee on Cancer

### Factors associated with the survival of spCRC patients

**Fig. 7:**
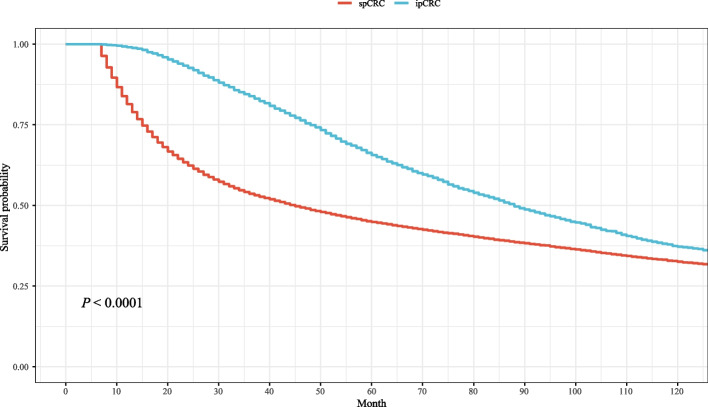
The survival curves of patients with ipCRC and spCRC

The survival status of patients with ipCRC and spCRC in our study were compared and we observed that patients with spCRC had poor survival probability than patients with ipCRC (Fig. 7). The data in Table 3 revealed that age, race, insurance, tumor site, histology, tumor grade, T stage, N stage, M stage, AJCC stage, radiation, chemotherapy, and radiation sequence might be associated with the mortality of ipCRC patients. In the multivariable cox regression model, we found that increase age (HR = 1.04, 95%CI: 1.04–1.04), Black (HR = 1.15, 95%CI: 1.13–1.17), uninsured (HR = 1.45 (1.42–1.47), insured/no specifics (HR = 1.13 (1.11–1.14), any Medicaid (HR = 1.10 (1.08–1.12), mucinous adenocarcinoma (HR = 1.06, 95%CI: 1.04–1.08), signet ring cell carcinoma (HR = 1.40 (1.34–1.46), higher tumor grade, higher T stage, higher N stage, higher M stage, high AJCC stage, radiation, chemotherapy, radiation before or after surgery were linked with higher risk of morality in ipCRC patients. Tumor site of left colon (HR = 0.94, 95%CI: 0.93–0.95) or rectum (HR = 0.88, 95%CI: 0.86–0.89) were correlated with lower risk of mortality in ipCRC patients. In terms of patients with spCRC, age, race, insurance, tumor site, histology, grade, T stage, N stage, M stage, AJCC stage, radiation, chemotherapy, and radiation sequence might be risk factor for the mortality of spCRC patients. Multivariable cox regression revealed that older age (HR = 1.02, 95%CI: 1.02–1.03), male (HR = 1.13, 95%CI: 1.04–1.23), Black (HR = 1.20, 95%CI: 1.06–1.35), uninsured (HR = 1.36, 95%CI: 1.16–1.59), signet ring cell carcinoma (HR = 1.64, 95%CI: 1.19–2.25), T4 stage (HR = 1.63, 95%CI: 1.32–2.01), N2 stage (HR = 1.36, 95%CI: 1.08–1.72), M1 stage (HR = 4.51, 95%CI: 2.00–10.18), AJCCIII (HR = 1.47, 95%CI: 1.08–1.98), and radiation (HR = 1.82, 95%CI: 1.43–2.33) were associated with increased risk of mortality in spCRC patients. Chemotherapy (HR = 0.94, 95%CI: 0.84–1.04), radiation after surgery (HR = 0.68, 95%CI: 0.47–0.81) or before surgery (HR = 0.55, 95%CI: 0.40–0.74) were correlated with decreased risk of mortality in spCRC patients.

**Table 3:** Factors associated with the mortality of ipCRC and spCRC

	ipCRC				spCRC			
	Model 1		Model 2		Model 1		Model 2	
	HR	*P*	HR	*P*	HR	*P*	HR	*P*
Age	1.04 (1.04–1.04)	< 0.001	1.04 (1.04–1.04)	< 0.001	1.02 (1.01–1.02)	< 0.001	1.02 (1.02–1.03)	< 0.001
Gender								
Female	Ref		Ref		Ref		Ref	
Male	1.00 (0.99–1.01)	0.999	0.88 (0.87–0.89)	< 0.001	1.05 (0.97–1.14)	0.236	1.13 (1.04–1.23)	0.003
Race								
White (ref)	Ref		Ref		Ref		Ref	
black	1.10 (1.09–1.12)	< 0.001	1.15 (1.13–1.17)	< 0.001	1.14 (1.01–1.29)	0.031	1.20 (1.06–1.35)	0.004
Other	0.83 (0.81–0.84)	< 0.001	0.86 (0.84–0.87)	< 0.001	-	-		
Unknown	0.13 (0.11–0.15)	< 0.001	0.18 (0.15–0.21)	< 0.001	0.86 (0.74–0.99)	0.033	0.89 (0.77–1.02)	0.100
Insurance								
Insured	Ref		Ref		Ref		Ref	
Uninsured	1.56 (1.54–1.59)	< 0.001	1.45 (1.42–1.47)	< 0.001	1.02 (0.77–1.36)	< 0.001	1.36 (1.16–1.59)	0.001
Insured/No specifics	1.16 (1.14–1.17)	< 0.001	1.13 (1.11–1.14)	< 0.001	1.10 (0.96–1.27)	0.1726	0.76 (0.69–0.83)	< 0.001
Any Medicaid	1.30 (1.28–1.32)	< 0.001	1.10 (1.08–1.12)	< 0.001	1.35 (1.19–1.57)	0.001	1.09 (0.94–1.25)	0.250
Unknown	1.28 (1.24–1.33)	< 0.001	1.45 (1.40–1.51)	< 0.001	0.73 (0.66–0.80)	0.730	1.22 (0.92–1.63)	0.167
Site								
Right colon	Ref		Ref		Ref		Ref	
Left colon	0.79 (0.78–0.80)	< 0.001	0.94 (0.93–0.95)	< 0.001	0.77 (0.67–0.89)	< 0.001	0.87 (0.74–1.02)	0.081
Others	2.81 (2.74–2.88)	< 0.001	1.35 (1.31–1.38)	< 0.001	0.89 (0.78–1.03)	0.113	1.12 (0.95–1.33)	0.184
Rectum	0.78 (0.77–0.79)	< 0.001	0.88 (0.86–0.89)	< 0.001	0.93 (0.61–1.41)	0.734	1.61 (0.86–3.01)	0.134
Histology								
Adenocarcinoma	Ref		Ref		Ref		Ref	
Mucous adenocarcinoma	1.14 (1.12–1.16)	< 0.001	1.06 (1.04–1.08)	< 0.001	0.91 (0.79–1.06)	0.226	0.88 (0.75–1.02)	0.084
Signet ring cell carcinoma	2.22 (2.13–2.32)	< 0.001	1.40 (1.34–1.46)	< 0.001	1.64 (1.20–2.24)	0.002	1.64 (1.19–2.25)	0.002
Others	0.71 (0.70–0.72)	< 0.001	0.85 (0.83–0.86)	< 0.001	0.80 (0.72–0.88)	< 0.001	0.93 (0.82–1.06)	0.259
Grade								
Grade I/Grade II	Ref		Ref		Ref		Ref	
Grade III/Grade IV	1.48 (1.45–1.51)	< 0.001	1.15 (1.11–1.18)	< 0.001	1.20 (1.08–1.33)	< 0.001	0.99 (0.89–1.11)	0.947
Unknown	2.01 (1.96–2.06)	< 0.001	1.27 (1.22–1.30)	< 0.001	0.95 (0.84–1.07)	0.367	1.05 (0.91–1.22)	0.507
T stage								
T1/T2	Ref		Ref		Ref		Ref	
T3	1.52 (1.50–1.55)	< 0.001	1.17 (1.14–1.20)	< 0.001	1.46 (1.33–1.61)	< 0.001	1.12 (0.93–1.37)	0.224
T4	3.25 (3.19–3.30)	< 0.001	1.76 (1.71–1.80)	< 0.001	2.23 (1.96–2.54)	< 0.001	1.63 (1.32–2.01)	< 0.001
Unknown	0.69 (0.66–0.72)	< 0.001	1.17 (1.14–1.20)	< 0.001	1.25 (1.09–1.44)	0.001	1.19 (0.85–1.67)	0.030
N stage								
N0	Ref		Ref		Ref		Ref	
N1	1.52 (1.50–1.54)	< 0.001	1.18 (1.15–1.21)	< 0.001	1.45 (1.31–1.61)	< 0.001	1.06 (0.85–1.32)	0.586
N2	2.45 (2.42–2.49)	< 0.001	1.80 (1.76–1.84)	< 0.001	1.91 (1.70–2.15)	< 0.001	1.36 (1.08–1.72)	0.010
N3	-	-	-	-	1.85 (0.69–4.94)	0.219	-	-
NX	1.81 (1.78–1.84)	< 0.001	1.05 (1.02–1.08)	< 0.001	1.07 (0.92–1.24)	0.382	1.86 (0.66–5.22)	0.241
M stage								
M0	Ref		Ref		Ref		Ref	
M1	5.54 (5.48–5.61)	< 0.001	3.00 (2.61–3.45)	< 0.001	2.44 (2.13–2.78)	< 0.001	4.51 (2.00–10.18)	0.001
MX	1.23 (1.21–1.26)	< 0.001	0.76 (0.73–0.79)	< 0.001	0.85 (0.73–0.99)	0.044	0.75 (0.52–1.08)	0.120
AJCC								
0	Ref		Ref					
I	1.20 (1.16–1.25)	< 0.001	1.14 (1.10–1.19)	< 0.001	Ref	-	Ref	
II	1.68 (1.62–1.75)	< 0.001	1.24 (1.18–1.30)	< 0.001	1.39 (1.24–1.55)	< 0.001	1.15 (0.93–1.43)	0.188
III	2.18 (2.09–2.27)	< 0.001	1.55 (1.48–1.63)	< 0.001	1.85 (1.65–2.07)	< 0.001	1.47 (1.08–1.98)	0.013
IV	9.31 (8.95–9.68)	< 0.001	1.84 (1.59–2.13)	< 0.001	3.08 (2.65–3.58)	< 0.001	0.61 (0.27–1.36)	0.227
Unknown stage	2.47 (2.37–2.57)	< 0.001	1.84 (1.74–1.94)	< 0.001	1.23 (1.06–1.43)	0.006	1.14 (0.79–1.63)	0.483
Radiation (No/Unknown)	1.06 (1.05–1.08)	< 0.001	1.04 (1.01–1.07)	< 0.001	1.23 (1.10–1.38)	< 0.001	1.82 (1.43–2.33)	< 0.001
Chemotherapy	0.85 (0.84–0.86)	< 0.001	1.47 (1.45–1.49)	< 0.001	1.29 (1.19–1.40)	< 0.001	0.94 (0.84–1.04)	< 0.001
Radiation sequence								
Only one	Ref		Ref		Ref		Ref	
After surgery	0.90 (0.88–0.93)	< 0.001	1.39 (1.33–1.44)	< 0.001	1.24 (1.06–1.46)	0.007	0.68 (0.47–0.81)	0.001
Before surgery	0.60 (0.58–0.61)	< 0.001	1.35 (1.31–1.40)	< 0.001	0.95 (0.77–1.16)	0.597	0.55 (0.40–0.74)	< 0.001
Both	0.77 (0.56–1.08)	0.128	1.17 (0.84–1.64)	0.347	0.44 (0.11–1.76)	0.246	0.37 (0.09–1.49)	0.160
Unknown	1.79 (1.55–2.05)	< 0.001	1.39 (1.20–1.60)	< 0.001	0.39 (0.06–2.78)	0.349	0.39 (0.05–2.80)	0.350

*spCRC* second primary colorectal cancer, *ipCRC* initial primary colorectal cancer, *AJCC* American Joint Committee on Cancer, *HR* hazard ratio, *CI* confidence interval

Model 1 Univariate cox regression analysis

Model 2 Multivariable cox regression analysis including variables with statistical difference in Model 1

## Discussion

This study evaluated the incidence of spCRC, factors associated with the occurrence of spCRC and survival of spCRC based on the data from SEER database. The results delineated that the incidence of spCRC was decreased on the whole. Age, gender, insurance, initial primary tumor site, histology, T stage, N stage, M stage, AJCC stage, and radiation sequence were factors associated with the risk of spCRC. The prognosis of spCRC patients was poor than ipCRC patients. Age, gender, race, insurance, histology, T stage, N stage, M stage, AJCC stage, radiation or chemotherapy, and radiation sequence were factors associated with the survival of spCRC patients.

Previous epidemiology studies have found that the trends of CRC were stabilizing or decreasing in highly developed countries including Canada and in Northern Europe [19]. Kanth et al. conducted a review on screening and prevention of CRC, which revealed that the incidence of CRC has been declining in some countries such as North America [20]. Herein, we found that the overall incidence of spCRC was decreased, this might be attributed to improved treatments of the initial primary cancers, as the initial primary cancers status were risk factors for spCRC [10]. A previous study from Bae et al. indicated that the occurrence of spCRC showed high rate in the initial 2–4 years following the diagnoses of initial primary cancers [15]. Appropriate and timely treatments for initial primary cancers might reduce the incidence of spCRC.

Emerging studies have observed that the incidence of CRC was increased in young patients [21]. Bailey et al. estimated that the incidence of colon and rectal cancers including initial primary cancer and second primary tumor might increase by 90% and 124%, respectively, for patients aged 20–34 years by 2030 [22]. Sung et al. indicated that one of the most alarming current healthcare issues is the rise in CRC incidence in individuals aged 20–49 years [23]. Another epidemiology study based on a large integrated health system observed a rising rate of CRC incidence in 18–49 years’ people [24]. These findings might give support of the findings in our study, showing that the incidence of spCRC was increased in 15–39 years during 2004–2006, and 2008–2009. Although the trends of incidence of spCRC was decreased during 2009–2016, the high incidence of spCRC in young people still requires attention. More and more young people tend to have bad habits such as smoking and sedentary lifestyle, which were identified to be risk factors for CRC. Incidence of spCRC varies in cancer patients with different characteristics and identifying a population who are at high risk of developing spCRC is essential for optimal surveillance and management of patients with cancers. For cancer patients at young age, more actively treatments were required and healthier lifestyles and habits were advocated. In the current study, age, treatments such as chemotherapy, gender, race, grade, TNM stage, and AJCC stage were identified to be factors associated with spCRC. These were supported by various previous studies, which proposed that older age [25], well-differentiated disease, SEER distant staging, and male gender [10], and Black people [26, 27] were risk factors for developing spCRC. For cancer patients who bear these characteristics, early screening of spCRC was needed.

Pruitt et al. demonstrated that patients with previous cancer generally had worse overall survival compared to those without [12]. Chen et al. compared the prognosis between patients with ipCRC and spCRC, and revealed that patients with spCRC had worse prognosis than patients with ipCRC [9]. These data provided evidence to the results of the present study, which found that the overall survival of spCRC patients was lower than ipCRC patients. Some other studies demonstrated that the prognosis of patients with spCRC were similar with those with ipCRC [28, 29]. This disagreement might because of different databases used in these studies. Considering SEER program was a nationwide database, more spCRC patients were analyzed in this study, which suggested that the results might be reliable.

Factors including race, histology, tumor grade, TNM stage and AJCC stage associated with the outcomes of ipCRC and spCRC were evidenced by various studies [30–32]. In the current study, increased age was associated with elevated risk of death in both ipCRC and spCRC patients. This was supported by several previous studies. A study of Mohd et al. depicted that age was correlated with the lymph node metastasis and tumor metastasis in CRC patients, which implied that age was a factor associated with the prognosis of CRC patients [33]. Another important finding in this study was that tumor site was a prognostic factor for ipCRC patients. We found that tumor sites at left colon and rectum were associated with decreased risk of mortality in ipCRC patients. There was evidence showing that sidedness of CRC influenced the risk of second primary gastrointestinal malignancies [34]. Previously, the differences of right-sided colon cancer and left-sided colon cancer were widely noted [35, 36]. Patients with left-sided colon cancers showed a higher sensitivity to bevacizumab treatment and had longer survival than those with right-sided colon cancers [37, 38]. On the other hand, Takamizawa et al. compared the role of primary tumor location in patients with colorectal liver metastasis, and found rectal cancer might have worse relapse-free survival and overall survival compared with left-sided colon cancer in patients with colorectal liver metastases who underwent hepatic resection [39]. The differences among left-sided colon cancer, right-sided colon cancer and rectal cancer might be because left-sided colon cancer have better prognostic markers such as p53, and NRAS mutations, which might provide references for offering timely interventions for those with high risk of poor prognosis [40]. These findings suggested that patients with ipCRC or spCRC with the primary tumor site at left colon, right colon or rectum should be considered distinct entities, and the interventions should be provided based on the tumor sites.

The present study analyzed the incidence of spCRC using the large sample size from SEER database. Cancer survivors registered in SEER database were followed-up, which could better evaluate the incidence of spCRC. Our analysis extended the current understanding of the risk of spCRC that cancer survivors faced. The estimation of the risk of spCRC among previous cancer cases might suggest the need for long-term follow-up surveillance for cancer patients and lead to patient-specific surveillance monitoring. The findings might also inform future targeted screening strategies among cancer survivors as well as help identify individuals who might benefit maximally from tertiary prevention strategies. Several limitations existed in the current study. Firstly, this was a retrospective study, patients with distant metastasis might be mistaken for spCRC, and we excluded patients with interval between the diagnosis of initial primary cancers and spCRC (latency) < 6 months, and histology being the same if the primary site is the same as the primary site of the initial primary cancers to reduce these errors. Secondly, variables including eating habits and living habits of patients such as smoking and drinking status, and genetic information were not included, which were reported to be associated with the risk of cancers [41–43], which might influence the results. The findings of our study still required validations in more studies.

## Conclusions

Our study used a large scale of data from SEER database to evaluate the incidence of spCRC, factors associated with the occurrence of spCRC and survival of spCRC based on the data from SEER database. We found that the incidence of spCRC was decreased except in people with initial primary tumor grade IV and those aged 15–39 years. The screening of CRC should be performed in more people at young age. The overall survival of spCRC patients was lower than ipCRC patients. Cancer patients with older age, high tumor grade, TNM stage, and AJCC stage should be caution to the occurrence of spCRC and timely interventions should be provided for spCRC patients to improve their outcomes. The findings might also inform future targeted screening strategies among cancer survivors, and suggested the need for long-term follow-up surveillance for cancer patients.

## Supplementary Information

**Additional file 1:** **Supplementary Figure 1.** The initial primary tumor sites of spCRC patients.

## Data Availability

The datasets generated and/or analyzed during the current study are available in the SEER database, https://seer.cancer.gov/.

## Abbreviations

CRC: Colorectal cancer
SPM: Second primary malignancies
ipCRC: Initial primary CRC
ICD-O: International classification of diseases for oncology
SEER: Surveillance, epidemiology, and end results
AJCC: American joint committee on cancer
APC: Annual percentage change
