# Supplementary figures and images for: Second primary colorectal cancer in adults: a SEER analysis of incidence and outcomes
Source: BMC Gastroenterol. 2023 Jul 26;23:253. doi: 10.1186/s12876-023-02893-2 (PMC10373234; doi:10.1186/s12876-023-02893-2)

Supplementary Figure 1 The initial primary tumor sites of spCRC patients.

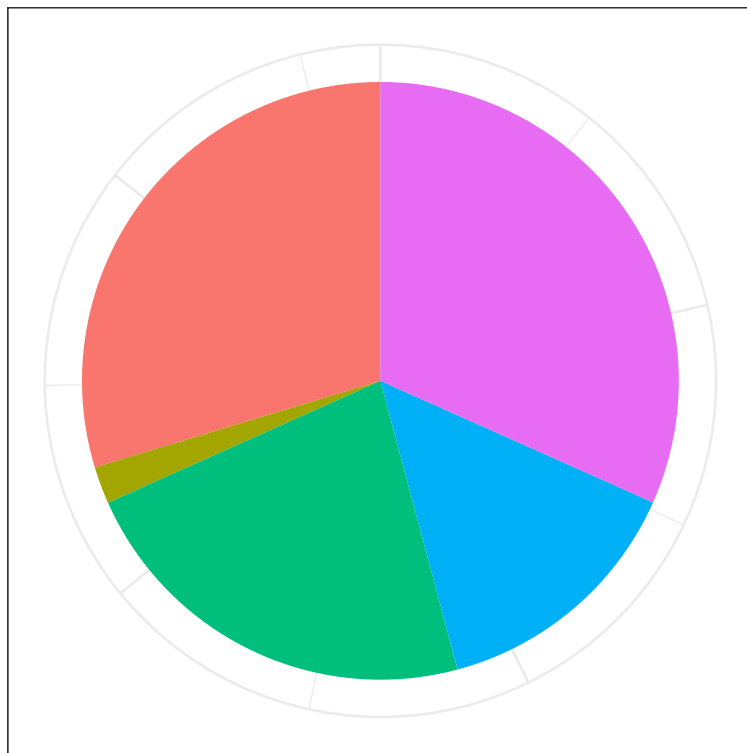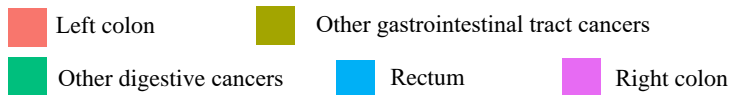

Supplement: Supplementary file 1 — Additional file 1: Supplementary Figure 1. The initial primary tumor sites of spCRC patients. [file 12876_2023_2893_MOESM1_ESM.pdf]
